# Analysis of proton pump inhibitor-associated drug problems and contributing factors among hospitalized nephrology patients

**DOI:** 10.3389/fphar.2026.1776655

**Published:** 2026-04-30

**Authors:** Ning Song, Xiaohong Zhang, Mei Han, Yuguang Guan

**Affiliations:** 1 Department of Pharmacy, Liangxiang Hospital of Beijing Fangshan District, Beijing, China; 2 Evidence-Based Medicine Center, Beijing University of Chinese Medicine, Beijing, China; 3 Department of Neurosurgery, Sanbo Brain Hospital, Capital Medical University, Beijing, China

**Keywords:** clinical pharmacist, drug-related problems (DRPs), influencing factors, nephrology, proton pump inhibitors (PPIs)

## Abstract

**Aims:**

To identify drug-related problems (DRPs) associated with proton pump inhibitors (PPIs) and their influencing factors among hospitalized nephrology patients, thereby providing evidence for the rational use of PPIs.

**Methods:**

A retrospective cohort of internal nephrology inpatients who received PPIs between June 2024 and May 2025 was assembled. Clinical pharmacists applied the PCNE classification system (version 9.1) to identify and categorize PPI-related DRPs. Univariate and multivariate logistic regression analyses were performed to explore factors contributing to inappropriate PPIs use.

**Results:**

A total of 520 patients were included, of which 241 patients (46.34%) experienced 303 DRPs. The most frequent problems involved drug selection and treatment duration. Multivariate analysis revealed that the following variables were independently associated with inappropriate PPIs use: purpose of PPI application (OR = 0.455, 95% CI 0.305–0.680, *p* < 0.001), antiplatelet agents (OR = 1.552, 95% CI 1.062–2.268, *p* = 0.023), anti-infective drugs (OR = 1.922, 95% CI 1.313–2.813, *p* = 0.001), and peritoneal dialysis (OR = 1.743, 95% CI 1.028–2.955, *p* = 0.039).

**Conclusion:**

Systematic identification of PPI-related DRPs and analysis of their determinants by clinical pharmacists will substantially promote the rational use of PPIs in hospitalized patients with renal disease.

## 1 Introduction

Proton pump inhibitors (PPIs) are among the most widely prescribed agents for acid-related disorders because they potently inhibit the gastric H^+^/K^+^-ATPase in parietal cells, providing reliable acid suppression with a relatively favorable safety profile. Their extensive use has made PPIs one of the top-ranked drug classes in hospital formularies worldwide (Ying et al., 2019).

Despite their therapeutic benefits, mounting evidence indicated that PPIs were frequently prescribed inappropriately for prolonged periods, higher doses or without a clear indication (Mohamed et al., 2024). A retrospective muti-centric study identified the most commonly (52.99%) prescribed potentially inappropriate medications (PIMs) in hospitalized older patients were PPIs using the 2023 Beers criteria (Prabahar et al., 2024). Such misuse not only contributed to the risk of adverse events, but also the economic burden.

In nephrology, patients with chronic kidney disease (CKD) had a higher prevalence of peptic ulcer disease, gastro-oesophageal reflux and the risk of gastrointestinal (GI) bleeding (Desbuissons and Mercadal, 2020). In addition, they were more likely to receive concomitant agents that may damage the gastric mucosa, such as glucocorticoids, antiplatelet drugs and non-steroidal anti-inflammatory drugs (NSAIDs) (Zhang et al., 2023). Consequently, PPIs were routinely employed in this population.

However, recent pharmaco-epidemiologic studies had highlighted a concerning link between PPIs exposure and renal injury. In 2016, two landmark studies connected PPIs to an excess risk for CKD (Freedberg et al., 2017). Moreover, emerging research by Chien et al. indicated that PPIs use was associated with an increased risk of progression to end-stage kidney disease (ESKD) in patients with pre-existing CKD compared with non-users. Notably, this risk increased substantially with the duration of PPIs therapy (Chien et al., 2025).

Furthermore, patients in nephrology department often presented with a high burden of comorbidities, including coronary heart disease, diabetes, hypertension, anemia, and depression, etc. (Albuhayri et al., 2022). This led to polypharmacy, which commonly included a range of medications, such as clopidogrel, iron supplements, calcium, sevelamer, antifungals, antidepressants, and immunosuppressants (Zhang et al., 2023). When co-administered with PPIs, these agents may engage in clinically drug-drug interactions (Wedemeyer and Blume, 2014) and affected efficacy. Given these complex factors, hospitalized patients in nephrology were typically at an elevated risk of DRPs involving PPIs and pharmacists should pay closer attention to whether PPIs were necessary and whether their usage was appropriate.

To date, few investigations had systematically characterized PPI-related DRPs in nephrology wards. This retrospective study therefore aimed to identify and classify PPI-related problems using the PCNE v9.1 (Pharmaceutical Care Network Europe, 2020) classification system, and then elucidate the contributing factors identified by clinical pharmacists in a comprehensive university teaching hospital serving the southwestern district of Beijing. The findings were intended to raise awareness among internal medicine physicians and promote rational use of PPIs in nephrology.

## 2 Methods

### 2.1 Setting and study design

Our study was registered on Chinese Clinical Trial Registry (ChiCTR2500111139) and approved by Ethics Committee of Liangxiang Hospital (2024035). Hospitalized patients in nephrology department (30-bed unit) at a tertiary hospital who received PPIs during the period from June 2024 to May 2025 were included. This also encompassed patients admitted to the renal intensive care unit (ICU). Patients taking self-provided oral PPIs or those with incomplete medical records were excluded.

Two clinical pharmacists, both holding clinical pharmacy training certificates issued by China Ministry of Health and possessing at least 5 years of hospital practice experience, independently identified PPI-related DRPs. In cases of discrepancy, Cohen’s kappa coefficient analysis was done on the evaluation results of two pharmacists and a senior pharmacist was consulted to reach a consensus. The evaluation criteria were based on drug instructions, Martindale pharmacology, clinical guidelines, and expert consensus. DRPs were identified through a pre-audit system and manual review of medical orders, focusing on improperly use in terms of indications, dosage, frequency, drug interactions and treatment duration. The PCNE-DRP v9.1 classification system was subsequently utilized to categorize these irrational usages, followed by an analysis of the underlying causes and influencing factors.

### 2.2 Data collection

All data were systematically retrieved from the Hospital Information System (HIS). Demographic characteristics included age, gender, smoking history and alcohol use history. PPIs usage data encompassed generic names, dosages, routes of administration, duration of treatment, and Defined Daily Doses (DDDs, the calculation formula for DDDs is total drug consumption in grams/DDD value in grams). Clinical information regarding history of ulcer, renal disease stages, presence of hemodialysis or peritoneal dialysis, surgical history, and concomitant medications (such as corticosteroids, anticoagulants, antiplatelets, anti-infectives, antidepressants, immunosuppressants, and chemotherapy drugs) were also collected.

### 2.3 Statistical analysis

Continuous variables were expressed as means and standard deviations (SDs) if normally distributed, or as medians with interquartile ranges (IQRs) otherwise. Categorical variables were presented as frequencies and percentages. A binary logistic regression analysis was conducted to identify independent risk factors associated with DRPs. Variance inflation factor (VIF) values were calculated to assess the degree of multicollinearity among the variables that were significant in the univariate analysis (*p* < 0.10). VIF values greater than 10 were considered to indicate multicollinearity, and such variables were excluded from the multivariate logistic regression analysis. The results were reported as odds ratios (ORs) with 95% confidence intervals (CIs). All statistical analyses were performed using the Statistical Package for Social Sciences (SPSS version 26.0). A *p*-value < 0.05 was considered statistically significant.

## 3 Results

### 3.1 Patient demographic and clinical characteristics

A total of 520 hospitalized patients receiving PPIs were enrolled in this study. The cohort comprised 254 males (48.8%) and 266 females (51.2%), with a mean age of 66.2 ± 13.3 years. Smoking history was documented in 193 patients (37.1%), while 147 patients (28.3%) reported alcohol consumption. Among the 520 patients, 353 (67.9%) received PPIs for therapeutic reasons, whereas 167 (32.1%) were prescribed them prophylactically. CKD was prevalent, affecting 405 participants (77.9%). Specifically, 338 (65%) patients were classified at stage 5, with 229 (44.0%) undergoing hemodialysis and 72 (13.8%) on peritoneal dialysis. The average length of hospital stay was 11.9 ± 3.4 days (Table 1).

**TABLE 1:** Patient demographic and clinical characteristics (n = 520).

Variables	Category	Value	DRP n (%)		*P*
		n (%)	No	Yes	
Gender	Male	254 (48.8)	123 (48.4)	131 (51.6)	0.028
	Female	266 (51.2)	155 (58.3)	111 (41.7)	
Age group	<60	128 (24.6)	76 (59.4)	52 (40.6)	0.174
	≥60	392 (75.4)	202 (51.5)	190 (48.5)	
Smoking history	No	327 (62.9)	175 (53.6)	152 (46.4)	0.974
	Yes	193 (37.1)	103 (53.4)	90 (46.6)	
Alcohol use history	No	373 (71.7)	206 (55.2)	167 (44.8)	0.199
	Yes	147 (28.3)	72 (49.0)	75 (51.0)	
History of ulcer	No	503 (96.7)	269 (53.5)	234 (46.5)	0.958
	Yes	17 (3.3)	9 (52.9)	8 (47.1)	
Purpose of PPI application	Prophylaxis	167 (32.1)	67 (40.1)	100 (59.9)	<0.001
	Therapy	353 (67.9)	211 (59.8)	142 (40.2)	
Chronic kidney disease stage	≤4	182 (35.0)	101 (55.5)	81 (44.5)	0.820
	5	338 (65.0)	177 (52.4)	161 (47.6)	
Hemodialysis	No	291 (56.0)	151 (51.9)	140 (48.1)	0.419
	Yes	229 (44.0)	127 (55.5)	102 (44.5)	
Peritoneal dialysis	No	448 (86.2)	247 (55.1)	201 (44.9)	0.057
	Yes	72 (13.8)	31 (43.1)	41 (56.9)	
Length of hospital stay	<10d	257 (49.4)	150 (58.4)	107 (41.6)	0.017
	≥10d	263 (50.6)	128 (48.7)	135 (51.3)	

### 3.2 Prevalence of DRPs

Among 520 hospitalized patients receiving PPIs, 241 patients (46.3%) were identified with a total of 303 DRPs, resulting in an average of 1.26 DRPs per patient. The result of Cohen’s kappa coefficient analysis was 0.75, indicating a high degree of consistency of the evaluation results of two pharmacists. The primary categories of DRPs were categorized as follows: “Effect of drug treatment not optimal on P1.2” (7 cases, 2.3%), “Adverse drug event (possibly) occurring on P2.1” (135 cases, 44.6%), and “Unnecessary drug treatment on P3.1” (161 cases, 53.1%). The prevalence and distribution of these DRPs were detailed in Table 2.

**TABLE 2:** Categories of PPI-related DRPs.

Categories of DRPs	Number of cases/case	Proportion/%
P1 Treatment effectiveness	7	2.31
P1.1 No effect of drug treatment despite correct use	​	​
P1.2 Effect of drug treatment not optimal	7	2.31
P1.3 Untreated symptoms or indication	​	​
P2 Treatment safety	135	44.55
P2.1 Adverse drug event (possibly) occurring	135	44.55
P3 Others	161	53.14
P3.1 Problem with cost-effectiveness of the treatment	​	​
P3.2 Unnecessary drug-treatment	161	53.14
Total	303	100

### 3.3 Distribution of causes for DRPs

To identify the underlying causes for these DRPs, a comprehensive analysis was conducted. A total of 327 specific causes were identified across the 303 DRPs, as a single DRP may arise from multiple factors. For instance, the DRP “P2.1 Adverse drug event (possibly) occurring” may be attributed to “C1.3 Inappropriate combination of drugs,” “No indication for drug” and “C4.2 Duration of treatment too long.” The most significant contributing factor was drug selection, encompassing issues such as “C1.1 Inappropriate drug choice,” “C1.2 No drug indication,” and “C1.3 Inappropriate combination of drugs.” Other prominent causes included “C4.2 Duration of treatment too long,” “C2.1 Inappropriate drug form,” and “C3.5 Dose timing instructions wrong, unclear or missing” (Table 3).

**TABLE 3:** Causes of PPI-related DRPs.

Categories of causes	Number of cases/case	Proportion/%
C1. Drug selection	210	64.22
C1.1 Inappropriate drug according to guidelines/formulary	32	9.78
C1.2 No indication for drug	104	31.80
C1.3 Inappropriate combination of drugs, or drugs and herbal medications, or drugs and dietary supplements	74	22.63
C2. Drug form	19	5.81
C2.1 Inappropriate drug form/formulation (for this patient)	19	5.81
C3 Dose selection	47	14.37
C3.5 Dose timing instructions wrong, unclear or missing	47	14.37
C4. Treatment duration	51	15.59
C4.2 Duration of treatment too long	51	15.59
Total	327	100

### 3.4 Influencing factors contributing for the occurrence of DRPs

To identify the influencing factors contributing to DRPs, both univariate and multivariate logistic regression analyses were conducted. In the univariate analysis, variables with a *p*-value < 0.10 were selected for further investigation. These included gender, purpose of PPI application, length of hospital stay, antiplatelet agents, anti-infective drugs, and peritoneal dialysis. The results of multicollinearity analysis showed that VIF values of six factors were less than 2, indicating that there was no significant multicollinearity in our model. Subsequently, these variables were introduced into a multiple logistic regression model. The results indicated that purpose of PPI application, antiplatelet agents, anti-infective drugs, and peritoneal dialysis retained statistical significance in the multivariate analysis (Table 4).

**TABLE 4:** Univariate and multivariate logistic regression of the factors associated with PPI-related DRPs among patients with kidney dysfunction.

Variables	COR (95% CI)	*P*	AOR (95% CI)	*P*
Gender	0.665 (0.472–0.939)	0.020	0.697 (0.482–1.010)	0.056
Age (years)	1.006 (0.993–1.019)	0.401	​	​
Smoking history	1.016 (0.711–1.452)	0.931	​	​
Alcohol history	1.303 (0.888–1.912)	0.176	​	​
History of ulcer	1.026 (0.390–2.703)	0.958	​	​
Purpose of PPI application	0.455 (0.313–0.663)	0.000	0.455 (0.305–0.680)	0.000
Length of hospital stay	1.014 (0.998–1.030)	0.084	1.003 (0.986–1.019)	0.743
Medicine use	​	​	​	​
Glucocorticoids	0.883 (0.486–1.607)	0.684	​	​
Antiplatelet agents	1.597 (1.129–2.260)	0.008	1.552 (1.062–2.268)	0.023
Dual antiplatelet drugs	1.032 (0.627–1.700)	0.900	​	​
Immunosuppressants	0.955 (0.405–2.252)	0.917	​	​
Anticoagulants	0.955 (0.550–1.659)	0.871	​	​
Anti-infective drugs	2.124 (1.495–3.017)	0.000	1.922 (1.313–2.813)	0.001
Antidepressants	0.607 (0.294–1.254)	0.178	​	​
Antiepileptic drugs	0.567 (0.169–1.908)	0.360	​	​
Operation	1.043 (0.606–1.794)	0.881	​	​
Stages of kidney disease	0.959 (0.883–1.040)	0.310	​	​
Hemodialysis	0.866 (0.612–1.226)	0.418	​	​
Peritoneal dialysis	1.625 (0.984–2.686)	0.058	1.743 (1.028–2.955)	0.039

## 4 Discussion

Although PPIs application for patients with kidney disease were still controversial, the DRPs associated with PPIs had garnered attention. Our research was the first to further systematically classify and analyze PPI-related DRPs in nephrology using the PCNE classification system.

The primary categories identified were “Effect of drug treatment not optimal,” “Adverse event (possibly) occurring,” and “Unnecessary drug treatment.” These findings aligned closely with those reported by Kyomya (Kyomya et al., 2023). Notably, “Unnecessary drug treatment” was the most prevalent DRPs (accounting for 53.1%), a proportion consistent with the commentary proportions noted in studies by Yadlapati (Yadlapati et al., 2017).

Ranking second in frequency, “Adverse drug event (possibly occurring)” constituted 44.6% of all DRPs encountered. Many studies had highlighted a strong link between PPI use and the onset of various pathological phenomena, including hip fractures, vitamin B12 defciency, hypomagnesaemia, ischaemic heart disease, stroke, pneumonia, acute and chronic renal dysfunction and *Clostridium*-related diarrhoea regardless of its format.

In our study, only 2.3% of DRPs were classified as “Effect of drug treatment not optimal.” This finding suggested that the majority of patients responded well to PPIs therapy. Furthermore, A survey on patients’ satisfaction with PPIs revealed no significant differences in overall satisfaction among the continuous, on-demand, and intermittent therapy groups (Huh et al., 2023).

The analyses of the causes revealed that drug selection constituted the largest proportion of issues, with “C1.2 No drug indication” being the most prevalent category. In our study, the primary reasons for oral PPIs overuse were the prevention of gastro-duodenal ulcers in low-risk patients; receiving steroids at low doses or undergoing antiplatelet therapy alone; NSAID or anticoagulant treatment without risk factors for GI injury; and the over-treatment of functional dyspepsia or misdiagnosis of acid-related disorders. These findings were consistent with the review by Savarino (Savarino et al., 2017). Conversely, irrational use of injectable PPIs was primarily observed in stress ulcer prophylaxis within non-intensive care units (Orelio et al., 2021) and upper GI bleeding prevention for patients with positive occult blood tests due to the high risk of GI bleeding associated with CKD (Desbuissons and Mercadal, 2020).

In terms of “C1.3 Inappropriate combination of drugs”, our analysis highlighted significant pharmacological interactions between PPIs and medications frequently used by patients with kidney disease (Carollo et al., 2024). On one hand, the prolonged acid-suppressing effect of PPIs led to elevated intragastric pH, which may impair the absorption of various drugs, including iron supplements, vitamin B12, azole antifungals, and mycophenolate mofetil. On the other hand, PPIs inhibited transporters such as CYP2C19, CYP3A4 and p-glycoprotein, which may alter the metabolism of other drugs. Notably, omeprazole and esomeprazole were potent inhibitors of CYP2C19, reducing the conversion of clopidogrel into its active metabolite. This, in turn, led to decreased metabolism of warfarin (Henriksen et al., 2015), cilostazol, and escitalopram, resulting in increased plasma concentrations. Furthermore, all PPIs inhibited CYP3A4, potentially increasing plasma levels of tacrolimus (Itagaki et al., 2002) and voriconazole. A critical physical incompatibility issue arose from the weak alkalinity of most injectable PPIs, which may neutralize acidic drugs like cefoperazone, necessitating tubing flushes before and after infusion to prevent precipitation.

Regarding “C3 Dose selection,” the primary issue identified was “C3.5 Dose timing instructions wrong, unclear or missing.” This was largely attributed to the fact that oral PPIs should be administered 0.5–1 h before meals to ensure they are converted from precursors into active drugs and exert their function on the proton pump activated after eating. However, these specific timing instructions were frequently absent from medical orders in our study.

Rare DRPs reasons fell under “C2.1 Inappropriate drug form/formulation.” For patients with dysphagia or requiring nasogastric tube administration, standard enteric-coated or sustained-release tablets/capsules were unsuitable, as crushing them would destroy the protective coating and render them ineffective in the acidic stomach environment. Therefore, only orally disintegrating dosage forms or multi-unit microcapsule systems were appropriate formulations for these specific populations.

In terms of “C4.2 Duration of treatment too long,” our findings suggested that this issue was primarily driven by two factors. First, although patients were diagnosed with reflux esophagitis or gastroesophageal reflux disease, PPIs were prescribed for durations exceeding guideline-recommended courses. According to a multicenter study, PPIs ranked as the second most common class PIMs, accounting for 12.63% of cases identified by the STOPP/START criteria. Specifically, PPIs were prescribed at full therapeutic doses for more than 8 weeks in older adults with uncomplicated peptic ulcer disease, without a valid long-term indication (Prabahar K., 2025). Second, prolonged use was also common among patients receiving intravenous PPIs for stress ulcer prophylaxis (Eisa et al., 2014). Clinically, patients should be reassessed after 3 days of therapy, and PPIs should be discontinued promptly once the stress-related risk had resolved. Although the standard treatment course typically ranges from 3 to 7 days, with a maximum recommended duration of 2 weeks, our study found that some patients received PPI therapy for over 3 weeks. This extended use may increase the risk of PPI-related adverse events, including hospital-acquired pneumonia (HAP) and *Clostridium difficile*-associated diarrhea (Desbuissons and Mercadal, 2020).

Furthermore, logistic regression analysis identified several influencing factors for PPI-related DRPs among nephrology inpatients, including purpose of PPI use, concurrent antiplatelet drugs, anti-infective agents, and peritoneal dialysis.

The results showed that patients receiving antiplatelet therapy were more likely to use PPIs primarily for the prevention of gastric mucosal injury caused by these agents. However, current clinical guidelines specify that PPIs co-administration was only recommended for patients with specific risk factors for GI bleeding (Targownik et al., 2022). These risk factors included advanced age, concurrent use of anticoagulants or NSAIDs, steroids use, and *H. pylori* (*Helicobacter pylori*) infection. Consequently, the primary reasons for DRPs in this context were identified as the lack of a valid medical indication and unnecessary combination therapy involving PPIs when these specific risk factors were absent.

The use of anti-infective drugs was identified as an associated factor for the inappropriate use of PPIs in stress ulcer prophylaxis. Guidelines from the American Society of Health-System Pharmacists (ASHP) specified that acid-suppressive therapy should be reserved exclusively for critically ill patients meeting specific high-risk criteria (MacLaren et al., 2025). These criteria included coagulopathy, prolonged mechanical ventilation (>48 h), history of GI ulcer or bleeding within the past year, sepsis, ICU stay exceeding 1 week, occult GI bleeding persisting for ≥6 days, or steroid therapy requiring >250 mg/day of hydrocortisone equivalent. However, many infected patients lacking these risk factors were still prescribed PPIs. This unnecessary combination therapy often led to adverse drug event (possibly), primarily manifesting as antibiotic-associated diarrhea and *Clostridium difficile* infection, which were attributed to gut dysbiosis caused by the concurrent use of antibiotics and PPIs.

The purpose of PPI application was strongly associated with overuse, primarily driven by excessive prophylactic use in the absence of valid indications. Out-of-indication prevention included (Dharmarajan, 2021): non-selective NSAID users without high-risk profiles for upper GI complications; COX-2 inhibitor users without a prior history of GI bleeding; antiplatelet therapy in patients lacking high-risk factors (age > 65 years, concomitant corticosteroids/anticoagulants, or peptic ulcer disease history; stress ulcer prophylaxis administered to non-critically ill hospitalized patients who were not at high risk for ulcer formation or GI bleeding.

In our study, peritoneal dialysis (PD) was identified as a significant influencing factor for PPI-related DRPs, which was consistent with previous findings (Tay et al., 2024). These studies concluded that patients undergoing chronic renal failure on dialysis exhibit high PPIs consumption and frequent long-term treatment due to the impact of PD on gastroesophageal reflux symptoms and dyspeptic symptoms (Walia et al., 2022). Therefore, the primary causes of PPIs overuse in this population were attributed to the lack of valid medical indications and excessively prolonged treatment durations.

Our study has several limitations. First, this was a retrospective, single-center study conducted at a non-specialized center where nephrology is not the primary department. Therefore, the patient sample size was relatively restricted, potentially limiting the generalizability of our findings. Second, while we evaluated the appropriateness of PPIs prescriptions according to clinical guidelines, subjective judgment may have influenced our assessment. Third, we did not account for the specific complications associated with renal patients in our analysis.

## 5 Conclusion

This study confirms that the application of PPIs is highly prevalent among inpatients in the nephrology department in China. However, DRPs associated with PPIs use were also common, primarily attributed to the lack of valid medical indications and excessively prolonged treatment durations. The results of multivariate logistic regression analysis identified several independent risk factors for inappropriate PPIs use, including purpose of PPI administration, concurrent use of antiplatelet drugs or anti-infective agents, and undergoing peritoneal dialysis. Our findings provide a critical basis for clinicians and clinical pharmacists to implement targeted interventions, thereby promoting the rational and evidence-based use of PPIs in nephrology practice.

## Data Availability

The raw data supporting the conclusions of this article will be made available by the authors, without undue reservation.

## References

[B1] Albuhayri A. H. Alshaman A. R. Alanazi M. N. Aljuaid R. M. Albalawi R. I. M. Albalawi S. S. (2022). A cross-sectional study on assessing depression among hemodialysis patients. J. Adv. Pharm. Technol. & Res. 13 (4), 266–270. 10.4103/japtr.japtr_322_22 36568049 PMC9784045

[B2] Carollo M. Crisafulli S. Selleri M. Piccoli L. L’Abbate L. Trifirò G. (2024). Agreement of different drug-drug interaction checkers for proton pump inhibitors. JAMA Netw. Open 7 (7), e2419851. 10.1001/jamanetworkopen.2024.19851 38980677 PMC11234238

[B3] Chien Y. F. Chen Y. Y. Wu C. K. (2025). Association of pneumonia, fracture, metabolic, and renal events with long-term proton pump inhibitor use in patients with chronic kidney disease. Pharmacother. J. Hum. Pharmacol. Drug Ther. 45 (8), 512–521. 10.1002/phar.70043 40678972

[B4] Desbuissons G. Mercadal L. (2020). Use of proton pump inhibitors in dialysis patients: a double-edged sword? J. Nephrol. 34 (3), 661–672. 10.1007/s40620-020-00808-y 32710264

[B5] Dharmarajan T. S. (2021). The use and misuse of proton pump inhibitors: an opportunity for deprescribing. J. Am. Med. Dir. Assoc. 22 (1), 15–22. 10.1016/j.jamda.2020.09.046 33321078

[B6] Eisa N. Bazerbachi F. Alraiyes A. H. Alraies M. C. (2014). Do all hospitalized patients need stress ulcer prophylaxis? Clevel. Clin. J. Med. 81 (1), 23–25. 10.3949/ccjm.81a.13070 24391103

[B7] Freedberg D. E. Kim L. S. Yang Y.-X. (2017). The risks and benefits of long-term use of proton pump inhibitors: expert review and best practice advice from the American Gastroenterological Association. Gastroenterology 152 (4), 706–715. 10.1053/j.gastro.2017.01.031 28257716

[B8] Henriksen D. P. Stage T. B. Hansen M. R. Rasmussen L. Damkier P. Pottegård A. (2015). The potential drug–drug interaction between proton pump inhibitors and warfarin. Pharmacoepidemiol. Drug Saf. 24 (12), 1337–1340. 10.1002/pds.3881 26395871

[B9] Huh C. W. Son N. H. Youn Y. H. Jung D. H. Kim M. K. Gong E. J. (2023). Real-world prescription patterns and patient satisfaction regarding maintenance therapy of gastroesophageal reflux disease: an observational, cross-sectional, multicenter Study. J. Neurogastroenterol. Motil. 29 (4), 470–477. 10.5056/jnm23088 37814437 PMC10577463

[B10] Itagaki F. Homma M. Yuzawa K. Fukao K. Kohda Y. (2002). Drug interaction of tacrolimus and proton pump inhibitors in renal transplant recipients with CYP2C19 gene mutation. Transplant. Proc. 34 (7), 2777–2778. 10.1016/s0041-1345(02)03409-7 12431607

[B11] Kyomya J. Atwiine F. Shegena E. A. Muhindo R. Yadesa T. M. (2023). Drug-related problems and associated factors among patients with kidney dysfunction at a tertiary hospital in southwestern Uganda: a prospective observational study. BMC Nephrol. 24 (1), 375. 10.1186/s12882-023-03437-2 38114948 PMC10731752

[B12] MacLaren R. Dionne J. C. Granholm A. Alhazzani W. Szumita P. M. Olsen K. (2025). Executive summary-society of critical care medicine and American Society of Health-System Pharmacists Guideline for the prevention of stress-related gastrointestinal bleeding in critically ill adults. Am. J. Health-System Pharm. 82 (17), e747–e750. 10.1093/ajhp/zxaf165 40719551

[B13] Mohamed M. R. Itani M. Abohelwa M. Ahmed M. A. Abdouni L. Doumat G. (2024). The silent epidemic: inappropriate use of proton pump inhibitors among hospitalized patients. Arab J. Gastroenterology 25 (4), 414–420. 10.1016/j.ajg.2024.07.001 39069424

[B14] Orelio C. C. Heus P. Kroese-van Dieren J. J. Spijker R. van Munster B. C. Hooft L. (2021). Reducing inappropriate proton pump inhibitors use for stress ulcer prophylaxis in hospitalized patients: systematic review of De-Implementation studies. J. General Intern. Med. 36 (7), 2065–2073. 10.1007/s11606-020-06425-6 PMC8298652 33532958

[B15] Pharmaceutical Care Network Europe (PCNE) (2020). The PCNE classification V9.1. Available online at: https://www.pcne.org/upload/files/449_2021-01-27_Report_online_validation_PCNE_classification_v9.1.pdf (Accessed October 5, 2020).

[B16] Prabahar K. (2025). Evaluation of potentially inappropriate prescriptions among the geriatric population in Tabuk, Saudi Arabia *via* the STOPP/START criteria, version 3: a multicentric Study. J. Eval. Clin. Pract. 31 (6), e70279. 10.1111/jep.70279 40975859

[B17] Prabahar K. Alhawiti M. Yosef A. Alqarni R. Sayd F. Yousuf S. (2024). Potentially inappropriate medications in hospitalized older patients in Tabuk, Saudi Arabia using 2023 beers criteria: a retrospective multi-centric Study. J. Multidiscip. Healthc. 17, 1971–1979. 10.2147/jmdh.S461180 38706504 PMC11070160

[B18] Savarino V. Dulbecco P. de Bortoli N. Ottonello A. Savarino E. (2017). The appropriate use of proton pump inhibitors (PPIs): need for a reappraisal. Eur. J. Intern. Med. 37, 19–24. 10.1016/j.ejim.2016.10.007 27784575

[B19] Targownik L. E. Fisher D. A. Saini S. D. (2022). AGA clinical practice update on De-Prescribing of proton pump inhibitors: expert review. Gastroenterology 162 (4), 1334–1342. 10.1053/j.gastro.2021.12.247 35183361

[B20] Tay H. Y. Islahudin F. Siaw Y. Y. Wong W. C. Mohd Tahir N. A. Firdaus Khan S. S. (2024). Drug-Related problems among peritoneal dialysis patients: a 12-Year retrospective cohort Study. Cureus 16, e69700. 10.7759/cureus.69700 39429412 PMC11490276

[B21] Walia N. Rao N. Garrett M. Yates K. Malone S. Holmes C. (2022). Proton‐pump inhibitor use and the risk of peritoneal dialysis associated peritonitis. Intern. Med. J. 53 (3), 397–403. 10.1111/imj.15601 34719853

[B22] Wedemeyer R.-S. Blume H. (2014). Pharmacokinetic drug interaction profiles of proton pump inhibitors: an update. Drug Saf. 37 (4), 201–211. 10.1007/s40264-014-0144-0 24550106 PMC3975086

[B23] Yadlapati R. Kahrilas P. J. (2017). When is proton pump inhibitor use appropriate? BMC Med. 15 (1), 36. 10.1186/s12916-017-0804-x 28219434 PMC5319103

[B24] Ying J. Li L.-C. Wu C.-Y. Yu Z.-W. Kan L.-D. (2019). The status of proton pump inhibitor use: a prescription survey of 45 hospitals in China. Rev. Española Enfermedades Dig. 111, 738–743. 10.17235/reed.2019.6155/2019 31373505

[B25] Zhang S. Zhang G. B. Huang P. Ren Y. Lin B. Shao Y. F. (2023). Drug-related problems in hospitalized patients with chronic kidney diseases and clinical pharmacist interventions.BMC Geriatr., 23 (1): 849. 10.1186/s12877-023-04557-y 38093184 PMC10717358

