# Correction: Analysis of proton pump inhibitor-associated drug problems and contributing factors among hospitalized nephrology patients

**DOI:** 10.3389/fphar.2026.1875101

**Published:** 2026-06-09

**Authors:** Ning Song, Xiaohong Zhang, Mei Han, Yuguang Guan

**Affiliations:** 1 Department of Pharmacy, Liangxiang Hospital of Beijing Fangshan District, Beijing, China; 2 Evidence- Based Medicine Center, Beijing University of Chinese Medicine, Beijing, China; 3 Department of Neurosurgery, Sanbo Brain Hospital, Capital Medical University, Beijing, China

**Keywords:** clinical pharmacist, drug-related problems (DRPs), inffuencing factors, nephrology, proton pump inhibitors (PPIs)

In the published article there was an error in section **2.2 Data collection**. In the section, the calculation formula for Defined Daily Doses was erroneously given as “DDD/100 patient-days”. The correct formula is “total drug consumption in grams/DDD value in grams”.

The original article has been updated.

